# 562 Influence of the COVID-19 Pandemic on Emergency Room Visits for Burn Injury

**DOI:** 10.1093/jbcr/irac012.190

**Published:** 2022-03-23

**Authors:** Sunny Gotewal, Shivan N Chokshi, Juquan Song, Amina El Ayadi, George Golovko, Steven E Wolf

**Affiliations:** University of Texas Medical Branch, Galveston, Texas; University of Texas Medical Branch at Galveston, Galveston, Texas; University of Texas Medical Branch, Galveston, Texas; University of Texas Medical Branch at Galveston, Galveston, Texas; University of Texas Medical Branch at Galveston, Galveston, Texas; University of Texas Medical Branch at Galveston, Galveston, Texas

## Abstract

**Introduction:**

The COVID-19 pandemic was a devastating occurrence that left millions in critical condition in emergency rooms (ER) across the country. While hospitalizations due to COVID-19 increased exponentially in the last year, several reports have indicated declines in ER use due to common non-COVID related problems. There is currently a dearth of literature examining the effect of the COVID-19 pandemic on emergency room use for acute burn injuries. Thus, we performed a retrospective database analysis using the TriNetX database to quantify the effects of COVID-19 on United States ER visits for acute burn injuries. We hypothesize that ER visits due to burn injury decreased, especially in patients with severe burn injuries- defined as burned total burn surface area (TBSA) >20%.

**Methods:**

Patients who visited the ER from 2010-2020 due to burn injury were identified using ICD-10 codes. We then stratified these patients by age (< 18 and ≥18), severe ( >20% TBSA) vs. non-severe (< 20% TBSA) burn injury, and by change over time in 1-year intervals from 2010 to 2020. Extracted data was analyzed using chi-square with p< .05 considered significant.

**Results:**

We identified a total of 24,620,393 ER visits from 2010-2020. Of these, 142,007 (0.58%) were due to burn injury. A large majority of burn-related ER visits were for non-severe burns (n=134,120, 94.4%). ER visits for acute burn injury decreased by 21.6% during 2020 when compared to years prior. Stratification by age group revealed that pediatric patients (< 18) had more significant decreases in ER Visits than adult patients (≥18). Pediatric patients visited the ER 71.6% less than adults during 2020. When stratified by burn severity, patients with severe burns ( >20% TBSA) and patients with non-severe burns (< 20% TBSA) had similar decreases in ER usage during 2020 when compared to years prior (21.7% and 24.6%, respectively). Further age analysis revealed that both pediatric patients with severe burns and pediatric patients with non-severe burns visited the ER less than their adult counterparts (71.4% and 60.9%, respectively). All of the above differences were statistically significant (p< .05).

**Conclusions:**

During the COVID-19 pandemic in 2020, there was a sharp decrease in ER usage by patients with severe and non-severe burn injuries. This decrease was particularly salient in pediatric populations across all TBSA data points measured.

